# Bringing MDMA-assisted therapy for PTSD to traditional healthcare systems: tending to set and setting

**DOI:** 10.3389/fpsyt.2025.1433999

**Published:** 2025-01-23

**Authors:** Dimitri Perivoliotis, Kayla Knopp, Shannon Remick, Allie Kaigle, Christopher S. Stauffer, Chandra Khalifian, Tamara R. Wachsman, Bettye E. Chargin, Andrew W. Bismark, Al Alam, Leslie Morland

**Affiliations:** ^1^ Mental Health, Veterans Affairs San Diego Healthcare System, San Diego, CA, United States; ^2^ Department of Psychiatry, University of California, San Diego, San Diego, CA, United States; ^3^ Departments of Psychiatry & Research, Veterans Affairs Loma Linda Healthcare System, Loma Linda, CA, United States; ^4^ Department of Pharmacy, Veterans Integrated Services Network (VISN) 23 Clinical Resource Hub, Eagan, MN, United States; ^5^ Mental Health, Veterans Affairs Portland Healthcare System, Portland, OR, United States; ^6^ Department of Psychiatry, Oregon Health & Science University, Portland, OR, United States; ^7^ Women’s Health Sciences Division, National Center for PTSD, Department of Veterans Affairs, Boston, MA, United States

**Keywords:** implementation, MDMA, MDMA-assisted therapy, psychedelics, psychotherapy, PTSD, veterans, Veterans Health Administration -VHA

## Abstract

Although effective evidence-based trauma-focused psychotherapies for posttraumatic stress disorder (PTSD) are available, a significant proportion of patients show a suboptimal response or do not complete them. MDMA-assisted therapy (MDMA-AT) for PTSD is a promising intervention currently being evaluated in numerous studies worldwide, including investigation for potential Food and Drug Administration (FDA) approval in the United States. The concepts of set and setting are foundational in psychedelic therapy and refer to the mindset a person brings to therapy and the environment in which it takes place, respectively. Both are believed to play a critical role in the individual’s experience and efficacy of MDMA-AT. In this article, we describe the importance of set and setting in MDMA-AT for PTSD and outline the advantages and challenges of implementing this novel intervention in large healthcare settings such as the Veterans Health Administration (VHA). Mostly derived from our experience conducting clinical trials of MDMA-AT for PTSD in VHA, we present specific and practical suggestions for optimizing set and setting from both the participant’s and clinician’s perspective in a manner that both leverages the opportunities of such settings and adapts to their challenges. These recommendations are intended to inform future MDMA-AT for PTSD research and, potentially, eventual clinical implementation efforts in traditional healthcare systems.

## 1 Introduction

Although the most effective PTSD psychotherapies produce large effect-size changes (1), many patients still experience clinically impairing PTSD symptoms, with VA’s National Center for PTSD estimating remission rates of around 50% (2). Many patients have expressed favorable views toward psychedelic-assisted therapies and strong support for further research (3, 4). MDMA (3,4-methylenedioxymethamphetamine) is a psychoactive drug broadly classified as a non-classical psychedelic (5), currently classified as an illegal Schedule 1 substance in the United States. The effects of MDMA can manifest in cognitive, emotional, somatic, and transpersonal domains. The drug has been shown to increase social behaviors (6) and disclosure of emotionally laden content (7), while increasing empathy (8) and openness (9). These effects on consciousness and mood are thought to underlie its potential to catalyze therapy, which has led to the development of MDMA-assisted therapy (MDMA-AT) for PTSD.

The therapeutic effects of psychedelics like MDMA are believed to result from an interaction between the drug and the psychological and environmental contexts in which it is taken. “Set” (mindset) refers to a patient’s intentions, expectations, and overall mental health, and “setting” is the environment in which the therapy occurs. The concepts of set and setting can be traced to shamanic psychedelic use by indigenous cultures, where the substances are typically delivered in a ritualized fashion (10, 11). Outcomes have been found to be significantly worse when set and setting are not considered or manipulated in a negative manner (12).

MDMA-AT sessions in clinical trials have mostly been conducted at academic research centers and private clinics. We propose there is an opportunity for traditional healthcare organizations to lead the way as systems of care for patients who need such innovative PTSD treatment options. However, guidelines for establishing a conducive set and setting in these systems are lacking, and although consistent documentation and reporting of set and setting-related variables is recommended for research trials (13), most recent publications do not address these components in detail.

In this paper, we address the benefits and challenges of conducting MDMA-AT in traditional healthcare systems and provide practical suggestions for establishing an optimal set and setting for both patients and clinicians based on our experience conducting clinical trials of MDMA-AT in one such system, the Veterans Healthcare Administration (VHA). Our guidance is intended to assist researchers wishing to conduct clinical trials of MDMA-AT and can be further applied in clinical settings where it is or becomes an approved treatment.

## 2 Importance of set and setting

Patients taking MDMA in a therapeutic setting tend to become more emotionally vulnerable, and previously avoided traumas may rise to the surface. Thought patterns often become more flexible and open, and people and items in the environment may influence what is remembered and shared. Physiological sensations tend to become amplified, and there is often increased sensitivity to sound, light, and touch. Interpersonal dynamics like transference, countertransference, rupture, and repair may also become amplified and accelerated in this context. It is therefore imperative for clinicians to practice cultural humility (14, 15) and optimize set and setting in an individualized manner that is conducive to the therapeutic process.

Efforts to optimize set before ingestion of a psychedelic drug like MDMA are important because the mindset the individual brings to the session can shape their response to the drug. The non-ordinary state of consciousness and perceptual changes induced by psychedelics can induce a sense of loss of control and anxiety and reflexive attempts to control or resist the experience. Yet, surrendering to the experience has been associated with a more positive subjective response and better outcomes (16). However, for some individuals, for example those with PTSD, being able to surrender or “trust the process” is not something that readily feels safe. MDMA can also amplify emotional states and psychological struggles. Such effects can be a therapeutic opportunity, but the individual may be distracted from addressing core underlying issues if they enter the session preoccupied with secondary stressors such as anxiety about the impending MDMA experience or routine stressors they encountered in the hours leading up to their session (e.g., work, traffic). Another challenge involves patient expectations that may reduce the credibility and acceptability of psychedelic interventions. For example, drug use is prohibited during military service, with many servicemembers’ only exposure to psychedelics emphasizing their perceived risks, harms, and illegal status.

The physical setting in which a psychedelic session occurs (e.g., light, music, decor, people present) can similarly shape subjective response and outcomes. For example, the subjective effects of MDMA tend to be stronger when it is taken in the presence of others vs. alone (17) and MDMA-AT is intended to take place in a comfortable, living-room like atmosphere with careful attention to furnishings, décor, and music to facilitate the drug’s effect on heightening sensory experience (13). Setting also includes the social, historical, and cultural context in which the psychedelic session takes place, which can in turn influence the patient’s set. For example, people of color may mistrust psychedelic therapies and research due to their history of exploitation in medical research and inequities in the criminal justice system for drug-related offenses (18, 19).

## 3 MDMA-AT for PTSD

**Figure 1:**
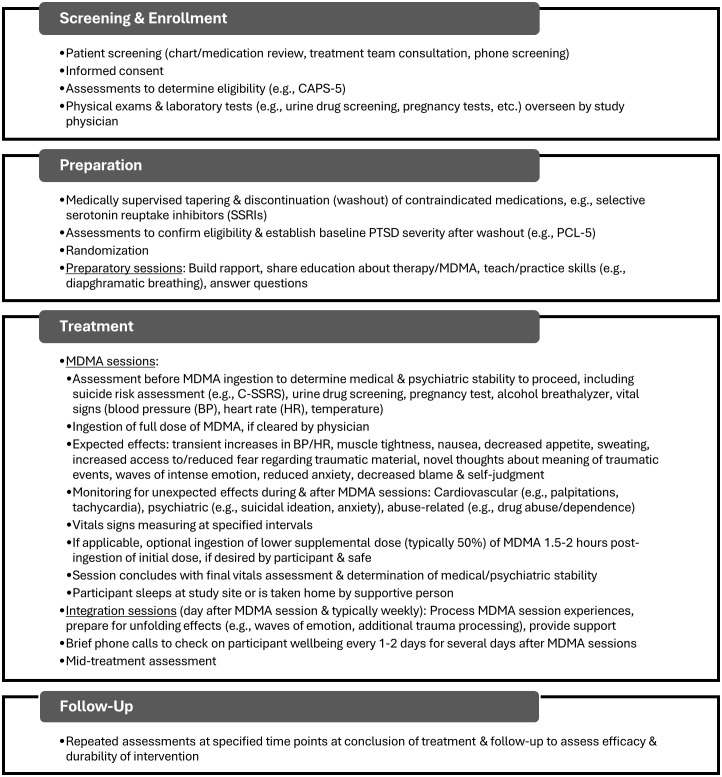
General overview of typical sequence of events in MDMA-AT drawn largely from the protocol used in Phase 3 clinical trials (20, 21) and our own studies (28–30). Specific procedures will vary depending on protocol and may change as research progresses. Certain steps presented (e.g., randomization) would not apply to clinical implementation. Additional details can be found in previously published protocols. CAPS-5, Clinician-Administered PTSD Scale for DSM-5 (31); PCL-5, PTSD Checklist for DSM-5 (32); C-SSRS, Columbia Suicide Severity Rating Scale (33).

Lykos Therapeutics (formerly the Multidisciplinary Association for Psychedelic Studies (MAPS) Public Benefit Corporation) has developed and evaluated a protocol for MDMA-AT for PTSD. In Phase 3 trials conducted thus far (20, 21), the protocol consisted of three 90-minute preparatory psychotherapy sessions, three 6-8-hour MDMA sessions spaced about a month apart, and a total of nine 90-minute integrative psychotherapy sessions following the MDMA sessions to process material that emerged during and after the latter and to help translate changes into sustained recovery (22). The therapy was conducted by a co-therapist team who took a stance of non-interference and empathic support, fostering patients’ emotional and physical safety to allow for inner-directed experience. **Figure 1** provides a general overview of the typical procedures in MDMA-AT for PTSD studies to date.

Phase 3 clinical trials of the intervention found a favorable risk-to-benefit profile as well as clinically significant reduction of PTSD symptoms and remission of the disorder (20, 21). These data were submitted to the FDA as part of a New Drug Application for MDMA used in combination with psychological intervention for adults with PTSD and the application received Priority Review in 2024. Ultimately, the FDA decided against approval, requesting another Phase 3 study with additional safety and efficacy data before reconsideration (23). The decision is anticipated to stimulate additional research on MDMA-AT in the coming years. In 2023, Australia became the first country to allow psychiatrists to prescribe MDMA for use in psychedelic-assisted psychotherapy to treat PTSD in controlled settings (24). In the United States, the state of Utah in 2024 authorized two large healthcare systems to offer behavioral health treatment programs for MDMA therapy for PTSD on a limited basis as part of a 3-year pilot program (25).

In 2021, the first dose of MDMA was administered within the VHA as part of a research study based on the MDMA-AT protocol examined in previous clinical trials. Presently, studies of that protocol and various adaptations are underway at six U.S. Dept. of Veterans Affairs (VA) healthcare facilities. In 2023, Congress approved an amendment to an appropriations bill directing VA to perform largescale psychedelic research (26). In 2024, VA issued a request for applications for proposals from VA researchers to study the use of psychedelic compounds in treating PTSD and depression (27); hence, the number of studies conducted in VA is anticipated to increase.

## 4 Implementation of MDMA-AT in healthcare settings

Large healthcare settings that provide integrated care alongside research and training programs make excellent venues to support MDMA-AT for PTSD. For example, as the largest healthcare system in the United States (34), VA plays an essential role in developing, testing, and disseminating innovative mental health treatments, particularly for PTSD. PTSD is prevalent among veterans, with a lifetime prevalence of approximately 23% among those who utilize VA healthcare (35). VA provides dedicated centers for training clinicians and supporting implementation of new treatments across its nationwide system of care. This system already supports the widespread use of evidence-based PTSD treatments recommended by VA/Department of Defense (DOD) Clinical Practice Guidelines (36). The interprofessional nature of integrated healthcare settings can also support the implementation of MDMA-AT; for example, our VA studies have involved collaboration across medical, psychological, peer support, and pharmacy services. Healthcare systems typically also offer access to emergency services, which may reduce risk and increase patient comfort.

At the same time, innovation within a traditional healthcare system comes with challenges (37). Both the practical logistics (e.g., 6-8-hour sessions with two therapists) and the philosophical stance (e.g., process guided by patients’ intuitive knowing, or “inner healer”) of MDMA-AT do not easily fit into existing models of mental health treatment, which often emphasize time-limited, therapist-directed, manualized, and skill-based scalable treatments. Often, treatment spaces are shared and décor is generic by design, resulting in a clinical or medical atmosphere. Attending to the set and setting surrounding the treatment for both patients and clinicians can help mitigate these barriers.

## 5 Optimizing the patient’s set

Optimizing the patient’s mindset begins during the preparation phase and continues throughout the intervention. For example, military servicemembers and veterans with PTSD often present with generalized mistrust as well as specific mistrust of the military and/or VA, and some patients may also experience mistrust of doctors, therapists, or medicines. Thus, a fundamental goal of preparation is to begin to earn the patient’s trust in the therapist team and the approach. Therapeutic alliance has been repeatedly shown to be a predictor of outcome in psychotherapy (38) and initial research suggests this is also the case in MDMA-AT (39). Therapists should ensure that patients understand the commitment of time and energy that is required for optimal outcome in this intensive intervention. Therapists should also appreciate the gravity of what is being asked of patients—to put their trust in an unconventional, intensive approach, two therapists they may not know well, and a drug they may have never taken, delivered in a system they may not fully trust. A patient’s decision to participate should be acknowledged as an important and courageous step toward their recovery, a process in which the therapists are privileged to take part. Furthermore, peer counselors can be beneficial team members, functioning as a trust-enhancing bridge between the patient and the clinical team (40).

A critical part of establishing set is expectation management. Given the immense media attention, patients are likely to have seen reports about psychedelics and MDMA-AT, some of which may be inaccurate. Asking what they already know can provide an opportunity to correct any misunderstandings and ensure informed consent.

Healthcare systems are evolving toward recovery-oriented models of care. For example, in VA’s Whole Health approach (41), the veteran is seen as an expert in their recovery, and treatment focuses not only on reducing symptoms but also improving functioning and quality of life. One principle of recovery is that patients have personal responsibility for their process of healing. Our medical culture can bias patients to approach psychedelic treatments with a “magic pill” mindset, placing undue emphasis on the drug versus their own role in the process. To counter this, therapists should emphasize and support the patient’s commitment to translating the effects of therapy into sustained behavioral changes in daily life.

Therapists and investigators should be appropriately hopeful yet careful not to oversell MDMA-AT. An essential component of informed consent in research or clinical settings is reviewing the limitations of the current knowledge base and possible risks in addition to benefits (42). There should be an explicit conversation about the possibility that the patient will not respond. Patients with chronic illness may seek psychedelic treatments after a string of unsuccessful traditional treatments, reporting being at the end of their rope. A nuanced conversation is needed about what exactly “getting better” looks like, emphasizing nonlinear and gradual change rather than sudden complete remission. Contingency plans in the event of suboptimal response should be made well before MDMA-AT begins.

A potential risk is that patients who experience less perceived improvement than expected may internalize this as personal failure. Throughout the therapy, encouraging patients to be patient, trust the process, and maintain mindful awareness of emotional, cognitive, and somatic changes that arise can help prevent them from blaming themselves and compounding already high levels of self-criticism and negative affect that accompany PTSD and depression. A central tenet of MDMA-AT is surrendering to the effects of the drug and being open to the unfolding experience, whatever that may be. Clinicians need to be aware of how challenging this expectation may be for their patients’ culture, e.g., in military operations, relinquishing control can mean extreme danger and potentially fatal consequences, so military servicemembers and veterans may require extra support with this aspect of the therapy.

The patient’s mindset can also be reinforced by tending to their support network. Educating referring clinicians about the rigors and potential outcomes of MDMA-AT can provide patients with an initial understanding of the intervention, and consultation with their other clinicians can reinforce growth and progress following its conclusion. Family members or other sources of social support deemed appropriately supportive by the patient and therapists should be included in the process if the patient is amenable. These individuals can support the patient’s mindset by helping to minimize psychosocial stressors and logistical demands on intervention days, and by helping to ensure the patient has a setting that is most conducive to healing (e.g., sleep, nutrition, quiet reflection) following therapy sessions. Given MDMA’s potential positive influence on social relationships (43), patients’ set may also be reinforced and validated by connecting them with peers (e.g., support groups, peer support specialists). For example, we have found that group integration can capitalize on the comradery that is naturally present in military culture, allowing veterans to cultivate a sense of belonging and acceptance, and support each other in their recovery process.

## 6 Optimizing the patient’s setting

Establishing a physical environment that is safe and therapeutic will promote the proper mindset to allow the patient to fully attend to their internal experience (13). Ideally, space would be designated within the facility for the primary purpose of conducting MDMA-AT. If this is not feasible, an alternative option could include a “pop-up” room approach where clinicians use portable supplies that are dismantled between sessions and stored in a locked closet.

The therapeutic environment should be in a quiet, private area where a 6-8-hour session can occur without interruption, in a room spacious enough for the therapists and patient to move around comfortably, as patients receiving MDMA may need to engage in movement and somatic expression. The room should include or be near necessary safety/monitoring equipment (e.g., blood pressure/heart rate monitor, thermometer, Automated External Defibrillator (AED)) and should be in close proximity to a private bathroom to accommodate potential increases in urination or gastrointestinal side effects such as nausea or vomiting without significant disruption to the session and to minimize the likelihood that patients will have to interact with anyone other than the therapists. Rooms should be furnished with comfortable furniture for both the patient (e.g., futon or couch that allows for lying down and sitting up) and therapists (chairs comfortable enough for all-day seating), a yoga mat for stretching, and adjustable non-fluorescent lighting and temperature. See **Table 1** for a list of recommended items for the room in which MDMA-AT is conducted but note that these may vary depending on local policies and research requirements.

**Table 1:** Recommended setup items for MDMA-AT sessions.

Safety	Décor
▪ Blood pressure and heart rate monitor ▪ Thermometer ▪ AED in close proximity	▪ Neutral art with calming visuals ▪ Plants and/or flowers ▪ Adjustable non-fluorescent lighting

Comfort & Experience	Electronics
▪ Sofa or futon ▪ Bedding, including blankets and pillows ▪ Eye mask ▪ Yoga mat and cushions ▪ Comfortable therapist seating ▪ Art supplies ▪ Healthy snacks ▪ Electrolyte fluids & water ▪ Food storage/refrigerator ▪ Culturally appropriate items to support experience and facilitate opening/closing of sessions, e.g., artwork, singing bowl, affirmation cards (22, 23)	▪ Laptop with webcam* ▪ Recording/storage platform (e.g., Teams, WebEx, Zoom)* ▪ External omnidirectional microphone* ▪ Stable Wi-Fi connection ▪ Mobile device (phone or tablet) for music ▪ Music subscription service (e.g., Spotify) ▪ Headphones ▪ Audio cables and splitter ▪ External speakers

Items may vary depending on institutional policies, local and federal regulatory requirements, and research vs. clinical application. *If recording sessions for fidelity assessment/supervision.

Adjustments can be made to the room to make it aesthetically pleasing and allow for reasonable personalization for each patient’s needs and cultural preferences. For example, inexpensive art with calming designs or nature scenes can be hung on the walls, and plants or fresh flowers (ensuring the patient is not allergic) added to the room. Culturally appropriate artwork and symbolism can be incorporated, e.g., one of our sites presents the MDMA capsule to the patient in a bowl handmade by another veteran in recovery from PTSD. We encourage patients to bring items that provide them with a sense of emotional comfort or reassurance or that can help prompt exploration of relevant traumatic material (e.g., sentimental, spiritual, or religious objects, photos), if indicated. The use of music to support the patient’s therapeutic experience is a vital element of psychedelic therapies—so much so that it has been called “the hidden therapist.” (44) The setting should permit music to be heard by the patient and therapists in the room and through headphones for the patient. Having other supplies available to help patients express and process their experiences may be helpful, including art supplies, aromatherapy, tactile objects, etc.

Patients should also be counseled to tend to the setting they are returning to at home, to ensure it is conducive to supporting any continued emotional processing that may unfold after MDMA sessions. Optimizing the setting of MDMA-AT extends to the patient’s interpersonal context, hence the importance of collaborating with patients’ partners and/or family, as described above.

## 7 Optimizing the clinician’s set & setting

Witnessing the powerful therapeutic changes that can take place in MDMA-AT can be rewarding for therapists, but the process can be cognitively, emotionally, and physically taxing because it demands a great deal of sustained focus and emotional presence. Hence, tending to set and setting is important for therapists as well as for patients.

Appropriate preparation for facilitating MDMA-AT includes formal training, supervised practice, and certification. Historically, psychedelic therapy training models have advocated for personal experience with psychedelics (45), but this presents legal and ethical concerns (46). Alternative, legal means of experiencing expanded states of consciousness (e.g., breathwork, meditation) have been suggested, but more research is needed to determine the utility of personal experience. The co-therapy team should ensure they feel comfortable and effective working with each other in this unique context and maintain frequent contact to support each other throughout the process. Ongoing consultation with other clinicians practicing MDMA-AT is also important for maintaining self-care and fidelity to the protocol.

Therapists are encouraged to pay close attention to appropriate self-care before, during, and after MDMA sessions, including getting adequate sleep, selecting comfortable chairs, doing what is needed to take care of their bodies (e.g., wearing comfortable clothes, stretching, taking breaks, eating), arranging for coverage to prevent being disturbed on MDMA session days, and being thoughtful about reducing other workload as needed. Therapists may need to educate their leadership about the unique nature of this work when designing schedules and workload expectations. This is not just a matter of good self-care and burnout prevention, but an ethical imperative, since burnout is associated with worse patient care outcomes (47). Likely due to the empathy-enhancing effects of MDMA, we have observed that patients attempt to take care of therapists who they sense are not taking care of themselves, which detracts focus from the patient’s own therapeutic process. When therapists are properly supported, MDMA-AT and other psychedelic-assisted therapies hold the potential to not only facilitate recovery in patients, but also to mitigate burnout in clinicians by expanding their repertoire of effective treatments and enhancing their self-efficacy.

## 8 Discussion

The concepts of set and setting are fundamental tenets of the therapeutic use of psychedelics that are believed to influence the effect of these substances. MDMA-AT for PTSD is a promising intervention currently being investigated in multiple studies. Given the current zeitgeist of increasing cultural openness to psychedelic treatments, this research is anticipated to increase and MDMA-AT to be re-considered for FDA approval in the U.S. We propose that traditional healthcare systems with integrated, recovery-oriented models of care would serve as ideal sites for the continued evaluation of MDMA-AT. As investigators of MDMA-AT research in one such system, the VHA, we have successfully navigated the challenges initially encountered, and present suggestions for optimizing set and setting from the perspective of both the patient and clinician for others pursuing the investigation or implementation of MDMA-AT in similar systems of care.

MDMA-AT research is a relatively complex endeavor. Specific guidelines on the nuances of conducting the intervention in routine healthcare settings—such as those presented here regarding set and setting—are therefore needed to ensure the rigor of future clinical trials, protect patient safety, and optimize outcomes. Moreover, if MDMA-AT is ultimately approved, there would likely be significant pressure to implement it in healthcare settings not designed for such treatments, which would require making the aforementioned adjustments to existing infrastructure, including specialized clinic spaces, extended session times, and adjusted workload requirements. Implementation of MDMA-AT in healthcare systems would also require addressing many other challenges beyond set and setting, such as complex regulatory and accreditation standards, insurance coverage and reimbursement, therapist training, etc., and doing so in a manner than preserves the integrity and maximizes the accessibility of these interventions (48). The decision by the FDA to not yet approve MDMA-AT could be an opportunity to develop strategies for successfully translating the intervention from the research space into the “real world.” In VA, recent and forthcoming studies include examining hybrid models that combine MDMA-AT with traditional trauma therapies (e.g., cognitive behavioral conjoint therapy (49) and prolonged exposure (50)), comparing its relative efficacy to traditional therapies (51), and group adaptation (30). MDMA and MDMA-AT may also be examined for conditions other than PTSD (52, 53).

One potential limitation of our suggestions for optimizing set and setting is that they are based largely on our clinical experience combined with our training in MDMA-AT, which itself is heavily inspired by the historical tradition of psychedelic-assisted psychotherapies. There is limited research supporting many of the suggestions made, and the underlying assumptions represent testable hypotheses, e.g., regarding the influence of expectation setting, the physical setting, having two therapists, etc. This kind of work has begun to take place in the field of psychedelics, for example, in efforts to establish evidence-based integration practices after psychedelic use (54) and to examine the impact of different types of music (55), but more research is needed specifically for MDMA-AT. The guidance we have presented is subject to change as we continue to learn about the intervention and its various models of implementation from current and future studies, but it is our hope that our suggestions can facilitate additional research and a greater understanding of the potential benefit of MDMA-AT.

## Data Availability

The original contributions presented in the study are included in the article/supplementary material. Further inquiries can be directed to the corresponding author.
